# Designing g-C_3_N_4_/PVP@Ca(OH)_2_ ternary heterostructure catalysts for efficient degradation of dyes, antibacterial activity, and molecular docking analysis

**DOI:** 10.1039/d5ra03570h

**Published:** 2025-09-15

**Authors:** Muhammad Ikram, Sawaira Moeen, Ali Haider, Iram Shahzadi, Anwar Ul-Hamid, Ghafar Ali, Asif Mahmood

**Affiliations:** a Solar Cell Applications Research Lab, Department of Physics, Government College University Lahore Lahore Punjab 54000 Pakistan dr.muhammadikram@gcu.edu.pk; b Department of Clinical Medicine, Faculty of Veterinary and Animal Sciences, Muhammad Nawaz Shareef, University of Agriculture Multan Punjab 66000 Pakistan; c School of Pharmacy, University of Management and Technology Lahore 54770 Pakistan; d Core Research Facilities, King Fahd University of Petroleum & Minerals Dhahran 31261 Saudi Arabia; e Nanomaterials Research Group (NRG), Physics Division, PINSTECH Islamabad 44000 Pakistan; f Centre for Clean Energy Technology, School of Mathematical and Physical Sciences, Faculty of Science, University of Technology Sydney City Campus, Broadway NSW 2007 Australia asif.mahmood@uts.edu.au

## Abstract

Global warming and environmental pollution demand urgent, sustainable solutions to mitigate their impacts on ecosystems and human health. To meet the rising need for efficient catalysts and antibacterial agents, advanced nanostructures have emerged as promising materials, offering enhanced functionality and sustainability in various applications. Here, we present a simple co-precipitation synthesis of ternary heterostructure (g-C_3_N_4_/PVP@Ca(OH)_2_) catalysts for catalytic dye degradation and bactericidal applications. The catalyst nanostructure is controllably synthesized by utilizing varying amounts of graphitic carbon nitride (g-C_3_N_4_) nanosheets anchored on a fixed quantity of PVP-capped Ca(OH)_2_ nanoparticles. Comprehensive characterization of the ternary heterostructure catalysts revealed polycrystalline behaviour, enhanced optical absorption, and decreased crystallite size. The modified g-C_3_N_4_/PVP@Ca(OH)_2_ heterostructures exhibited enhanced surface area, improved charge transfer efficiency, a large number of active sites, and increased stability. These attributes resulted in effective catalytic reduction of both coloured and colourless dyes and notable antibacterial activity against *Escherichia coli* (*E. coli*), supported by molecular docking analysis. The ternary g-C_3_N_4_/PVP@Ca(OH)_2_ heterostructure catalyst exhibited superior efficiency in degrading colored dyes compared to colorless compounds. Additionally, computational studies indicated the potential inhibitory effect of the synthesized catalyst on the DNA gyrase enzyme of *E. coli*. These findings highlight the promise of g-C_3_N_4_/PVP@Ca(OH)_2_ nanostructures as multifunctional materials for environmental remediation and antibacterial applications, underscoring the need for further investigation and optimization.

## Abstract 2

Global warming and environmental pollution demand urgent, sustainable solutions to mitigate their impacts on ecosystems and human health.

## Graphical abstract

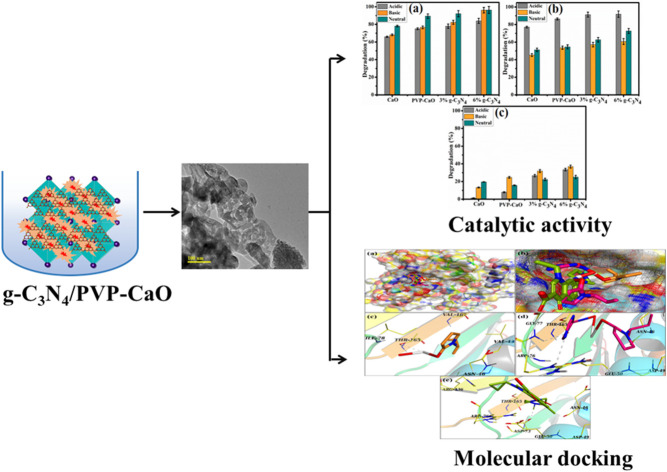

## 1. Introduction

The semiconductor catalysis method for water purification has emerged as a potential advanced technique to reduce environmental pollutants.^1^ Recently, Ca-based catalysts have gained interest for their usage in the fields of dye reduction and antibacterial activity.^2,3^ The essential use of Ca-based catalysts in the above mentioned fields is due to their non-toxicity, high stability, large surface area, non-corrosiveness, high porosity, and biocompatible behavior.^4,5^ Ca-based materials, including CaTiO_3_, CaFe_2_O_4_, CaMg(CO_3_)_2_, and Ca(OH)_2_, have been studied owing to their exceptional dye reduction properties.^6–9^ Nevertheless, the effectiveness of these specific catalysts is greatly influenced by the quick recombination of excitons.^1^ To date, Ca-based catalysts can be altered by structural doping with metals, composites with non-metals, and the development of heterojunctions with other catalysts to increase charge transfer potency.^1,10–12^ Among such advances, heterojunction development is a significant technique to decrease the recombination rate of excitons. Several Ca-based binary heterojunction catalysts, including CaO/SrTiO_3_, CaTiO_3_/g-C_3_N_4_, and CaMoO_4_/CaWO_4_, have been investigated for considerable charge transfer potency and increased dye reduction ability.^12–14^

Recent photocatalysis or dye reduction advances include the systematic structure of a ternary system, which has shown promising results in environmental purification.^15^ The ternary system exhibits an extended surface area, high charge transfer potency, and increased optical absorption relative to a binary system.^16,17^ In the recent past, several ternary materials Ag–ZnO/CaO, ZnO/MgO/CaO, CaO/MgO/g-C_3_N_4_, and Zr–TiO_2_/CaO, have been synthesized for the reduction of dyes.^18–21^ Thus far, the conventional method for preparing ternary materials has entailed a multistep hydrothermal procedure, which significantly restricts the ability to scale up the synthesis process.^1^ Alternatively, co-precipitation is a facile, low-temperature, and scalable approach for the synthesis of ternary materials.^22^ Ca-based nanostructures (NSs) possessed a considerable bandgap energy (*E*_g_) of ∼5.54 eV, utilized in catalysis, biomedical, and antibacterial applications.^18,23^ Furthermore, the antimicrobial potency of Ca(OH)_2_ was ascribed to the generation of Ca^2+^ ions, which leads to cell death as it penetrates through bacterial membranes. Antibacterial properties of Ca(OH)_2_ were attributed not only to the production of reactive oxygen species (ROS) on their surface but also to the elevation of pH due to hydration, leading to the creation of hydroxides and the release of Ca^2+^ ions. These factors collectively impact the growth of the *E. coli* pathogen.^19^ Developing a ternary system with materials, as mentioned above, is an effective approach to enhance surface area and stability for dye reduction and bactericidal activity compared to the single and binary system.

In this work, we have developed a co-precipitation route for the preparation of facile ternary g-C_3_N_4_/PVP@Ca(OH)_2_ heterostructure materials with enhanced catalytic activity towards rhodamine B (RhB), methyl orange (MO), and benzoic acid (BA) dyes. The PVP is chosen as it is non-toxic and biocompatible, used as a capping agent to regulate the crystal growth, increase the surface area and stabilize the Ca(OH)_2_.^20,21^ Furthermore, the N atoms in the polyvinylpyrrolidone (PVP) backbone can improve the charge transfer from core Ca(OH)_2_ particles to the outer g-C_3_N_4_ layer. The g-C_3_N_4_ is utilized because of its large surface area and small *E*_g_, which is suitable for catalytic reduction and bactericidal behavior.^24^ The g-C_3_N_4_/PVP@Ca(OH)_2_ heterostructure exhibits a porous structure with high surface area, which offers large sites for interfacial contact. Moreover, the presence of different species in the g-C_3_N_4_/PVP@Ca(OH)_2_ heterostructure delivers high catalytic activities in diverse environments and a wide pH range. To the best of our knowledge, this is the first study to report ternary g-C_3_N_4_/PVP@Ca(OH)_2_ heterostructure for the degradation of dyes and antibacterial activity.

## 2. Experimental section

### 2.1 Materials

Calcium chloride dihydrate (CaCl_2_·2H_2_O, 99%), PVP [(C_6_H_9_NO)_*n*_, *M*_w_ 40 000], urea (CH_4_N_2_O), and NaOH were procured from Sigma Aldrich.

### 2.2 Synthesis of g-C_3_N_4_

g-C_3_N_4_ was prepared based on previous work through the pyrolysis of urea.^25^ A sufficient quantity of urea was promptly subjected to a furnace at 550 °C for 5 hours. This temperature converted urea into melamine, yielding a white powder of g-C_3_N_4_.

### 2.3 Synthesis of g-C_3_N_4_/PVP@Ca(OH)_2_

**Fig. 1:**
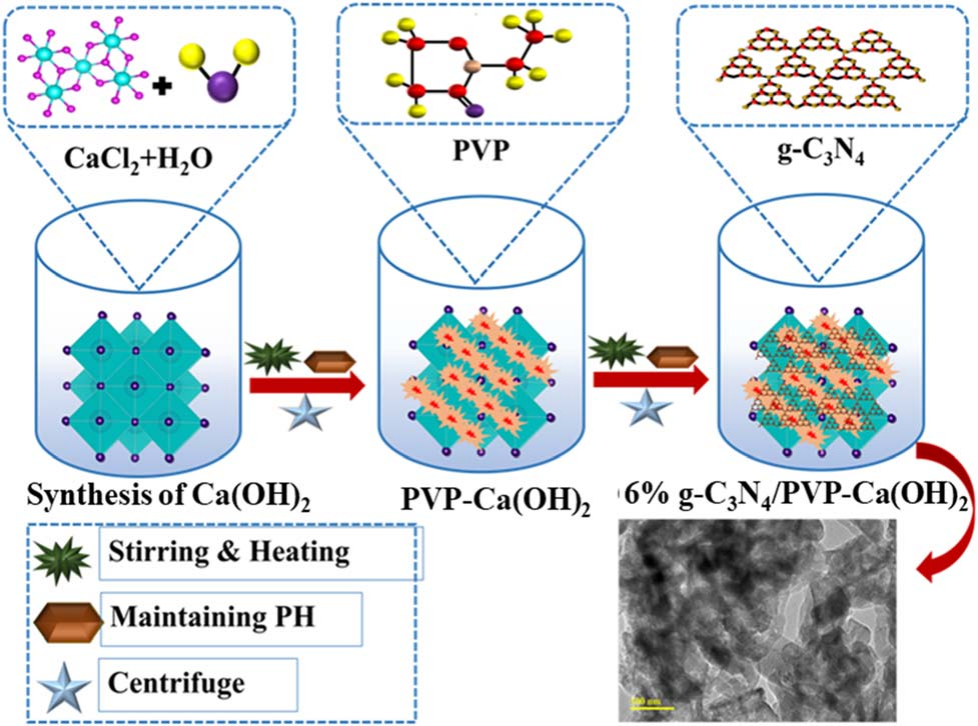
Synthesis of g-C_3_N_4_/PVP@Ca(OH)_2_ heterostructure.

To synthesize Ca(OH)_2_, initially, 0.5 M of CaCl_2_·2H_2_O was prepared under vigorous agitation at 80 °C. The precipitating agent (NaOH) was added to an agitated solution to form precipitates and maintain a pH of ∼10. Subsequently, the colloidal solution was centrifuged (8000 rpm, 8 min) repeatedly, vaporized overnight at 130 °C, and crushed into fine powder to achieve Ca(OH)_2_ nanostructures (sample 1). To prepare PVP@Ca(OH)_2_ nanostructures, 3 wt% PVP was added to the CaCl_2_·2H_2_O solution before the addition of NaOH to maintain the pH. After half an hour of stirring at 80 °C, the obtained solution was centrifuged, dried at 130 °C, and ground into fine powder (sample 2). Following a similar procedure, a set quantity of g-C_3_N_4_ was added to the precursor solution of PVP-Ca(OH)_2_ to prepare 3 and 6% g-C_3_N_4_/PVP@Ca(OH)_2_ (Fig. 1). This named as samples 3 and 4 in the subsequent text. Few characterization of sample 1 were obtained from our previous work.^19,26^

### 2.4 Catalytic performance

The catalytic performance of Ca(OH)_2_, PVP@Ca(OH)_2_, and (3, 6 wt%) g-C_3_N_4_/PVP@Ca(OH)_2_ was analyzed using RhB and MO as colored dyes and BA as a colorless compound. The experiment was performed in the absence of light. The NaBH_4_ was added as a reducing agent. Separate solutions of dyes were prepared under continuous agitation and divided into neutral (pH ∼ 7), acidic (pH ∼ 3), and basic (pH ∼ 11) media, respectively. Initially, 0.1 M of reductant (NaBH_4_) was added separately into 3 mL solutions of RhB, MO, and BA dyes. In the next step, 400 μL of prepared ternary heterostructure catalysts were integrated into the above solutions (NaBH_4_ + dyes). The absorption/electronic spectra of catalysts were examined using a UV-vis spectrophotometer at regular intervals.

### 2.5 Differentiation and screening of MDR *E. coli*

#### 2.5.1 Sample acquisition and recognition of bacteria

Unpasteurized milk specimens were obtained from lactating cows at several locations in Punjab, Pakistan, including markets, farmlands, and veterinary clinics. Upon collecting, the milk was promptly delivered to the laboratory while maintaining a temperature of 4 °C. The occurrence of *E. coli* in unpasteurized milk was assessed by culturing three samples on MacConkey agar (MA) and incubating them at 37 °C for two days. Using gram staining and Bergey's Manual of Determinative Bacteriology biochemical tests, *E. coli* was identified.

#### 2.5.2 Antibacterial effectiveness

Ten distinctive MDR *E. coli* strains obtained from mastitic milk were examined for antibacterial potency of Ca(OH)_2_ and g-C_3_N_4_/PVP@Ca(OH)_2_ using well well-diffusion technique. MA Petri plates were cultivated with 0.5 McFarland MDR *E. coli* bacteria. Ca(OH)_2_, PVP@Ca(OH)_2_, and (3, 6%) g-C_3_N_4_/PVP@Ca(OH)_2_ were introduced into 6 mm wells on MA plate utilizing a purified cork borer at various doses as 0.5 and 1.0 mg/50 μL. The control medicine (0.005 mg/50 μL) consisted of ciprofloxacin, whereas the opposing control included (50 μL) DI water (deionized water). The extent of inhibition was quantified using a Vernier scale after colonization at a temperature of 37 °C for one day.

### 2.6 Molecular docking analysis

The study aimed to perform molecular docking on DNA gyrase from *E. coli* in relation to nucleic acid production pathways. The PDB ID used 5MMN, with a resolution of 1.90 Å. 3D structures were obtained from Protein Data Bank by matching the PDB IDs. The Sybyl X-2.0 software is employed to estimate molecular docking by constructing ligand structures. The water molecules with their inherent ligands were extracted, polar hydrogen atoms were introduced, and energy was conserved. Pymol was used to generate a three-dimensional model of binding interactions.^27–29^

### 2.7 DFT studies/MESP/HOMO/LUMO analysis

This theoretical framework enables discernment of electronic structures in atoms and molecules through integration of essential parameters, notably optimized geometries, frontier molecular orbital (FMO) energies, global and local reactivity descriptors, and molecular electrostatic potential (MESP) maps. Estimates were performed utilizing the B3LYP functional alongside SVP basis set, as executed in Gaussian 09 software (Revision E.01), adhering to established methodologies. The resultant checkpoint files underwent analysis through utilization of GaussView 6.^30,31^

## 3. Results and discussion

The g-C_3_N_4_/PVP@Ca(OH)_2_ ternary heterostructures were designed and synthesized through a carefully controlled process aimed at achieving multifunctional catalytic performance, as illustrated in Fig. 1. The CaCl_2_·2H_2_O was first converted into Ca(OH)_2_ followed by addition of PVP to form the PVP@Ca(OH)_2_ composite structures. The PVP was added because of its ability to stabilize the composite and promote uniform dispersion of the Ca(OH)_2_, which is essential for maximizing interactions with dye molecules during the catalytic process. To enhance the functionality of these composites, g-C_3_N_4_ was incorporated in varying amounts (4% and 6%), resulting in heterostructures with improved structural integrity and enhanced electronic and optical properties. The addition of g-C_3_N_4_ led to an improvement in the photocatalytic properties of the heterostructures, enabling effective degradation of dyes by promoting superior light absorption and efficient charge separation. The resulting g-C_3_N_4_/PVP@Ca(OH)_2_ ternary heterostructure exhibited excellent antibacterial activity due to synergistic effect of the heterostructure components. This controlled synthesis methodology provided excellent control over the composition and morphology of the resulting heterostructure for enhanced activities.

### 3.1 Structural properties

**Fig. 2:**
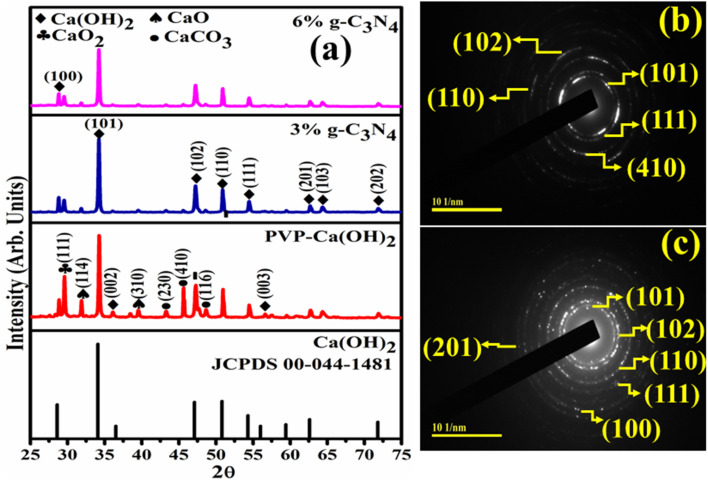
(a) Diffraction patterns of Ca(OH)_2_, PVP@Ca(OH)_2_, 3% g-C_3_N_4_/PVP@Ca(OH)_2_, and 6% g-C_3_N_4_/PVP@Ca(OH)_2_ (b and c) SAED images of PVP@Ca(OH)_2_ and 6% g-C_3_N_4_/PVP@Ca(OH)_2_.

The crystallite size, surface area and phase composition of the Ca(OH)_2_ and g-C_3_N_4_/PVP@Ca(OH)_2_ were characterized by the XRD (Fig. 2a). The diffraction peaks of Ca(OH)_2_ at 28.6° (100), 34.1° (101), 36.5° (002), 47.1° (102), 50.8° (110), 54.3° (111), 56.0° (003), 62.6° (201), 64.3° (103) and 71.8° (202) ascribed to the hexagonal phase (*P*3̄*m*1, no. 164), confirmed by the (JCPDS card no. 00-044-1481). Diffraction peaks at 31.9° (114) and 39.5°(310), were indexed to the cubic phase (*Fm*3̄*m*, no. 225) of CaO (ICDD card no. 00-017-0912).^32,33^ Bragg peak sited at 29.3° (111) confirmed the tetragonal lattice (*F*4/*mmm*) of CaO_2_ (JCPDS card no. 01-085-0514). Other reflection peaks at 43.1° (230), 45.4° (410), and 48.5° (116̄) correspond to the hexagonal lattice (*P*3_1_, no. 144) of CaCO_3_ (JCPDS card no. 01-083-1923, 00-047-1743). With PVP addition, overlapping of minor peaks observed between 25 to 30° was ascribed to the semicrystallinity of the capping agent (PVP).^34^ The relative intensity of the PVP-doped system was decreased, attributed to the enhancement in structural instability.^35^ The integration of g-C_3_N_4_ in a binary system (PVP@Ca(OH)_2_) leads to the shifting of peaks towards a lower angle as well as a reduction in the crystallinity. Compared to Ca(OH)_2_, the crystallite size was reduced from 27.4 to 17.2 nm for 6% g-C_3_N_4_/PVP@Ca(OH)_2_.

Additionally, SAED patterns consisting of identifiable circular rings were assigned to (101), (111), (410), (102), (110), (100), and (201) planes, well harmonized with diffraction patterns (XRD) (Fig. 2b and c).

### 3.2 Optical properties

**Fig. 3:**
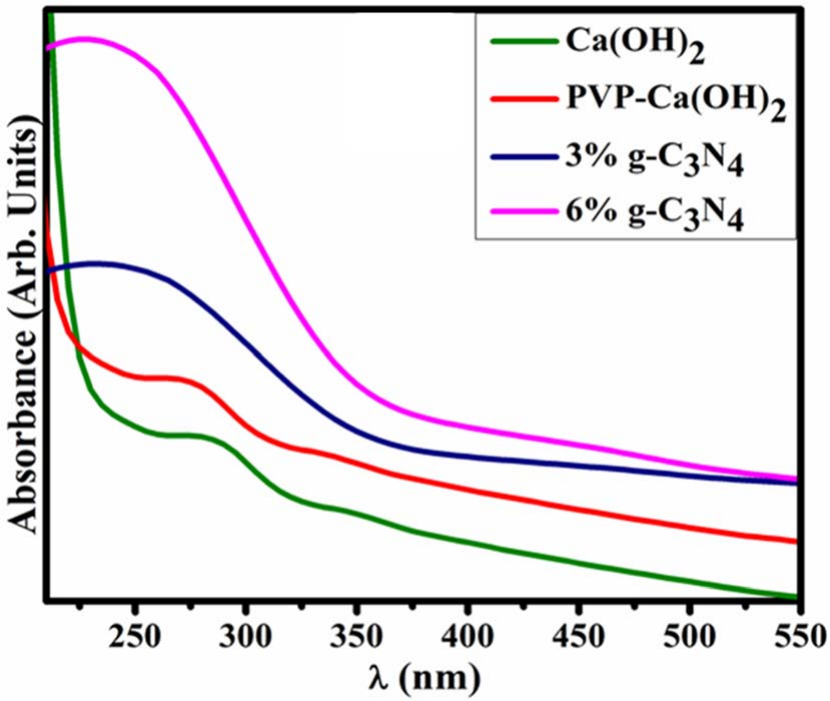
UV-vis spectra of Ca(OH)_2_, PVP@Ca(OH)_2_, 3%g-C_3_N_4_/PVP@Ca(OH)_2_, and 6% g-C_3_N_4_/PVP@Ca(OH)_2_.

The UV-vis spectroscopy was employed to investigate the optical properties and electronic structure of Ca(OH)_2_ and doped g-C_3_N_4_/PVP@Ca(OH)_2_ (Fig. 3). The absorption spectrum of Ca(OH)_2_ revealed a broad range of absorption between 200 and 800 nm, with the onset of absorption observed around 286 nm, attributed to the π–π* transition.^23,36^ In comparison, the binary and ternary products demonstrated increased absorption intensity accompanied by a bathochromic shift, likely caused by the presence of additional defect sites.^37^ Furthermore, the respective bandgap energies were calculated using the equation *E*_g_ = 1240/*λ*_onset_.^38^ The calculated *E*_g_ values for Ca(OH)_2_, PVP@Ca(OH)_2_, 3% g-C_3_N_4_/PVP@Ca(OH)_2_, and 6% g-C_3_N_4_ of PVP-Ca(OH)_2_ were 4.33, 4.29, 4.13, and 4.0 eV, respectively. These results highlight the progressive reduction in the bandgap with increased g-C_3_N_4_ doping, demonstrating its impact on enhancing the optical and electronic properties of the g-C_3_N_4_/PVP@Ca(OH)_2_ heterostructures.

### 3.3 Morphological properties

**Fig. 4:**
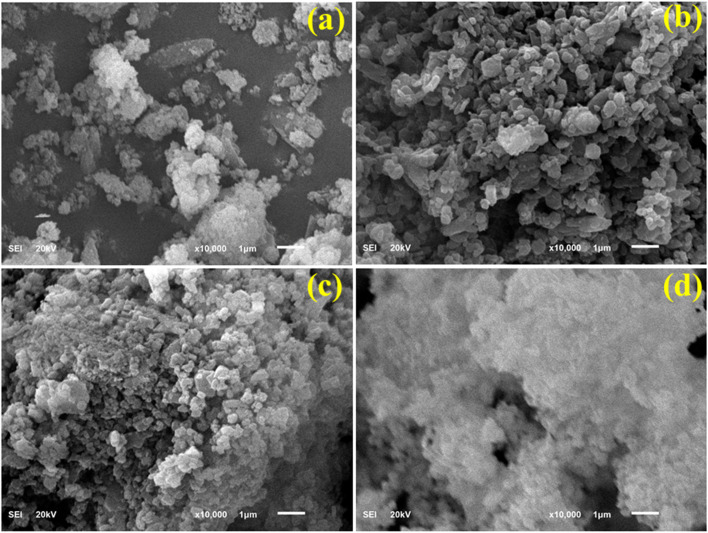
(a–d) FESEM images of (a) Ca(OH)_2_, (b) PVP@Ca(OH)_2_, (c) 3% g-C_3_N_4_/PVP@Ca(OH)_2_, and (d) 6% g-C_3_N_4_/PVP@Ca(OH)_2_.

The surface morphology and the porosity of the designed nanostructures play a critical role in determining the catalytic and antibacterial activities. The surface morphology for Ca(OH)_2_ and other derived products (*e.g.* 3 and 6% g-C_3_N_4_/PVP@Ca(OH)_2_) was thoroughly investigated using FESEM analysis, as shown in Fig. 4a–d. The FESEM analysis of Ca(OH)_2_ revealed a particulate morphology with multiple and irregular clusters of Ca(OH)_2_ nanoparticles, as shown in Fig. 4a. A structural rearrangement was observed upon addition of PVP, where well-defined particles in the size range of 200–300 nm were observed (Fig. 4b). It can be assumed that PVP acts as a dispersant for the Ca(OH)_2_ nanoparticles, where highly dispersed Ca(OH)_2_ nanoparticles were observed in PVP@Ca(OH)_2_ nanocomposites. Upon addition of g-C_3_N_4_, a slightly further reduction in particle size was observed as shown in Fig. 4c. Upon an increase in g-C_3_N_4_ concentration to 6%, not much difference in morphology was observed (Fig. 4c and d). The elemental composition of Ca(OH)_2_, PVP@Ca(OH)_2_, and (3, 6%) g-C_3_N_4_/PVP@Ca(OH)_2_ was confirmed through EDS analysis (Fig. S1a–d). Ca and O peaks verified the synthesis of Ca(OH)_2_, and the carbon (C) peak confirmed the addition of dopants (PVP, g-C_3_N_4_). Furthermore, EDS mapping of 6 wt% g-C_3_N_4_ sample exhibited the existence of Ca, O, and C through distinct colors (Fig. S2).

**Fig. 5:**
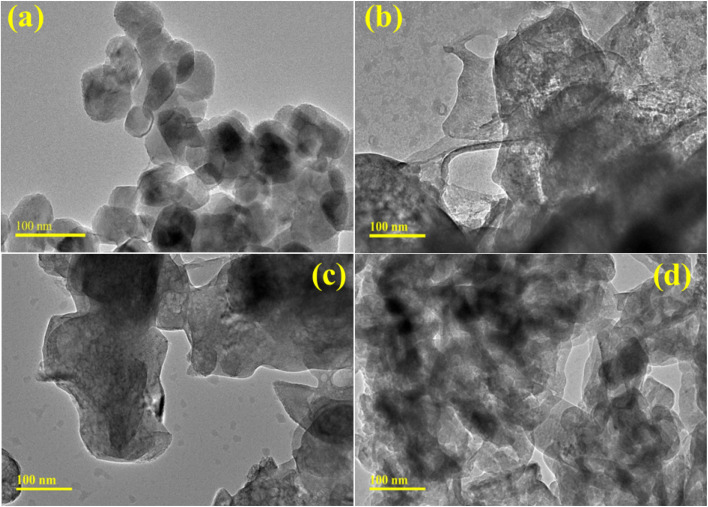
(a–d) TEM images of (a) Ca(OH)_2_, (b) PVP@Ca(OH)_2_, (c) 3% g-C_3_N_4_/PVP@Ca(OH)_2_, and (d) 6% g-C_3_N_4_/PVP@Ca(OH)_2_.

The TEM analysis was used to further examine the morphological properties of the Ca(OH)_2_ and g-C_3_N_4_/PVP@Ca(OH)_2_. The TEM analysis of Ca(OH)_2_ exhibited particle-shaped, randomly oriented, and interconnected nanoparticles with an average particle size of around 80–100 nm, as shown in Fig. 5a.^19^ To study the lattice structure of the Ca(OH)_2_, higher resolution TEM (HRTEM) analysis was carried out and presented in Fig. S3. The HRTEM analysis revealed a *d*-spacing of ∼0.21 nm, which correlates with the XRD peaks. The TEM analysis of PVP@Ca(OH)_2_ showed that the product still retains its particulate morphology but exhibits the presence of polymer in the product (Fig. 5b). The PVP covered Ca(OH)_2_ nanoparticles and formed a chain-like structure, acting as a bridge for the transportation of excitons between nanoparticles. Upon adding 3% g-C_3_N_4_, a nanosheet-like structure with numerous grooves formed, endorsing the overlapping with a binary system (PVP and Ca(OH)_2_), and this trend increased with higher concentrations (6%) of g-C_3_N_4_ (Fig. 5c, d and S3b). These interactions of g-C_3_N_4_ with Ca(OH)_2_ provide a large number of active sites for the catalytic decolorization of dyes.

### 3.4 Catalytic activity

**Fig. 6:**
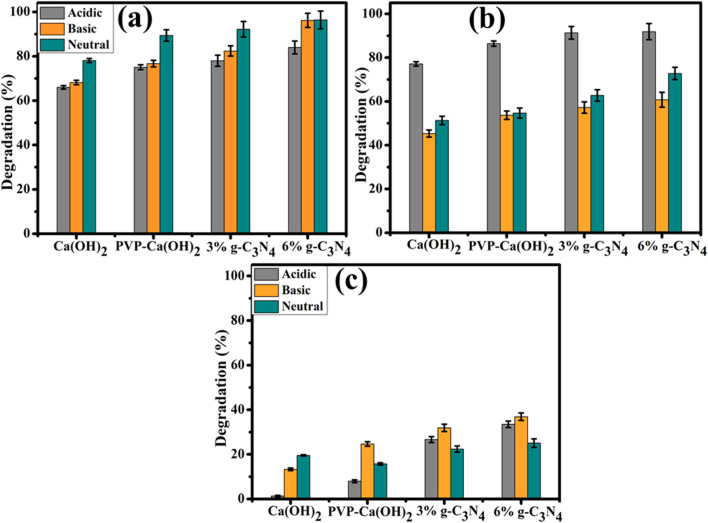
(a–c) Degradation of (a) RhB, (b) MO, and (c) BA in various media.

The degradation of colorful and colorless dyes by prepared catalysts was investigated through a UV-vis spectrophotometer (Fig. 6a–c). The catalytic mechanism for the reduction of colored dyes (MO, RhB) was observed in the presence of prepared heterostructure catalysts and reductant (NaBH_4_). The dye solution with reductant (NaBH_4_) alone without a catalyst does not show any degradation. The absorption intensity of MO (wavelength ∼ 460 nm), RhB (wavelength ∼ 554 nm), and BA (wavelength ∼ 220 nm) remains the same for several hours.^39–41^ Therefore, for reduction of colored and colorless dyes requires an effective catalyst along NaBH_4_. During catalysis, prepared heterostructure catalysts function as an electron transfer medium that transfers electrons from NaBH_4_, which acts as a donor (reducing agent), to colorful dyes (RhB and MO), which act as acceptors (oxidizing agent). This process causes the conversion of colorful dyes into the colorless compound.^42,43^

To investigate the efficiency of the developed catalysts, dye degradation was studied across a diverse pH range, including acidic, alkaline, and neutral solutions. Among the catalysts tested, the heterostructure with 6% g-C_3_N_4_ exhibited the best catalytic activity. When evaluating the degradation of RhB dye, the 6% g-C_3_N_4_/PVP@Ca(OH)_2_ catalyst proved superior across all pH ranges. In acidic media, it achieved an 84% degradation efficiency; in alkaline media, it reached 96.2%; and in neutral media, it exhibited 96.4% degradation efficiency, consistently outperforming other catalysts. In acidic media, the 6% g-C_3_N_4_/PVP@Ca(OH)_2_ demonstrated superior MO degradation, achieving an activity of 91.8%, significantly outperforming Ca(OH)_2_, which had an activity of 77.1%. In alkaline media, the 6% g-C_3_N_4_/PVP@Ca(OH)_2_ also excelled, with MO degradation activity reaching 60.7%, compared to 45.3% for Ca(OH)_2_. In neutral media, where degradation is generally more challenging, the 6% g-C_3_N_4_/PVP@Ca(OH)_2_ catalyst still exhibited remarkable activity, achieving 72.7% MO degradation, significantly higher than the other catalysts tested. The superior performance can be attributed to the increased surface area, enhanced porosity, and more active sites provided by the doping of graphitic carbon nitride, as confirmed by XRD analysis. For the colorless Benzoic Acid (BA) dye, the 6% g-C_3_N_4_/PVP@Ca(OH)_2_ catalyst showed the best performance in basic conditions with a maximum degradation of 36.9%; however, its performance in acidic and neutral media was less effective, highlighting the challenges associated with degrading colorless dyes. Overall, the 6% g-C_3_N_4_/PVP@Ca(OH)_2_ catalyst demonstrated exceptional catalytic efficacy, particularly for colored dyes like RhB and MO. The BET surface area of Ca(OH)_2_ and 6% g-C_3_N_4_/PVP-Ca(OH)_2_ was found to be 2.1130 ± 0.2757 and 2.7305 ± 0.2074 m^2^ g^−1^, respectively (Table S1). Comparison of the catalytic activity of the present work with the literature was added in Table S2.

### 3.5 Antibacterial activity

The antibacterial properties of Ca(OH)_2_, PVP@Ca(OH)_2_, and (3%, 6%) g-C_3_N_4_/PVP@Ca(OH)_2_ were evaluated, and the results are tabulated in Table 1. The inhibition zones for MDR *E. coli* ranged from 1.90 to 6.65 mm at the lowest doses (0.5 mg/50 μL) and from 2.25 to 9.65 mm at the highest doses (1.0 mg/50 μL). These results were compared to DI water (0 mm) and ciprofloxacin, which had an inhibition zone of 5.35 mm. The 6% g-C_3_N_4_/PVP@Ca(OH)_2_ catalyst exhibited the highest antibacterial activity, attributed to several synergistic mechanisms. It can be assumed that catalyst enhanced production of reactive oxygen species (ROS), such as hydroxyl radicals, hydroperoxyl radicals, superoxide anions, and hydrogen peroxide, which induced oxidative stress, damaging bacterial lipids, proteins, and DNA, and leading to cell death. Additionally, the physical interaction between the catalysts and bacterial cell walls can lead to membrane disruption and cell lysis. Furthermore, the formation of oxygen nanobubbles enhances this effect by creating internal pressure within the cells. The 6% g-C_3_N_4_/PVP@Ca(OH)_2_ nanostructures trap bacterial cells, preventing their proliferation, while the interruption of glycolysis hampers energy production. Furthermore, the release of metal ions disrupts bacterial enzymes and proteins. These combined effects collectively lead to superior antibacterial efficacy of the 6% g-C_3_N_4_/PVP@Ca(OH)_2_ catalyst, highlighting its potential for effective antibacterial applications.^44–48^ Comparison of the antibacterial activity of the present work with the literature was added in Table S3.

**Table 1:** Inhibition zones of Ca(OH)_2_ and doped Ca(OH)_2_ towards *E. coli*

Samples	Inhibition zone (mm) (0.5 mg/50 μL)	Inhibition zone (mm) (1.0 mg/50 μL)
Ca(OH)_2_	1.90	2.25
PVP@Ca(OH)_2_	5.40	7.90
3% g-C_3_N_4_	6.05	8.85
6% g-C_3_N_4_	6.65	**9.65**
Ciprofloxacin	5.35	**5.35**
DI water	0	**0**

**Fig. 7:**
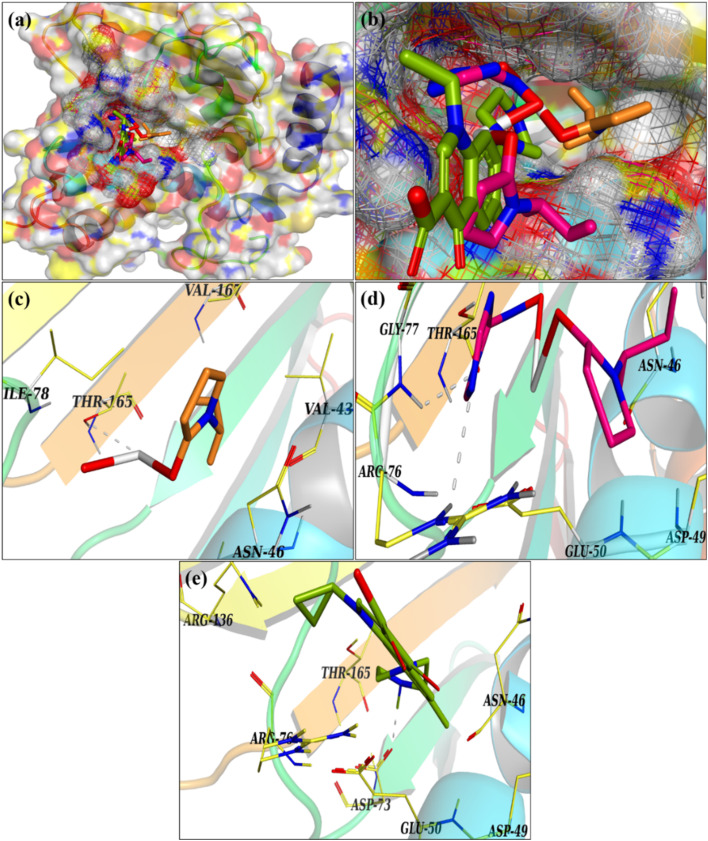
(a and b) 3D view of binding interaction of nanocomposites within the active site of DNA gyrase *E. coli* (c) PVP doped Ca(OH)_2_ (d) g-C_3_N_4_/PVP doped Ca(OH)_2_, (e) ciprofloxacin.

The microbicidal potential of nanoparticles containing metal ions and their interactions with bacteria through electrostatic, van der Waals, or hydrophobic forces has garnered significant research interest.^49,50^ To better understand these interactions at the molecular level, a molecular docking analysis was conducted, which provides more insights into how nanomaterials interact with bacterial enzyme targets, providing insights into their potential mechanisms of action. The molecular docking studies revealed that the modified nanomaterials have the capacity to interact with active site residues of specific enzyme targets, such as DNA gyrase (Fig. 7a and b). The g-C_3_N_4_/PVP@Ca(OH)_2_ demonstrated moderate affinities for DNA gyrase, indicating significant interactions with fundamental amino acids. The docked complexes formed hydrogen bonds with residues such as Thr165 (PVP-doped Ca(OH)_2_) and Gly77, Arg76, Glu50, and Thr165 (g-C_3_N_4_/PVP@Ca(OH)_2_), achieving binding scores of 2.16 and 3.45, respectively, as shown in Fig. 7c and d. These interactions suggest that the complexes can inhibit DNA gyrase, similar to the standard drug Ciprofloxacin, which has a binding score of 5.29 (Fig. 7e). The results of the molecular docking analysis are consistent with the observed antibacterial activity against *E. coli*, indicating that g-C_3_N_4_/PVP-doped Ca(OH)_2_ is a promising candidate for inhibiting bacterial growth. These results clearly demonstrate the efficiency of the developed catalysts.

**Fig. 8:**
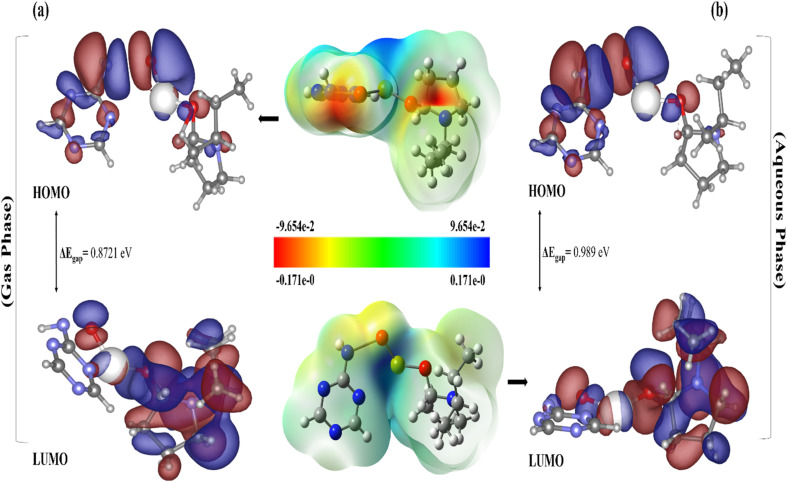
MESP and HOMO–LUMO analysis of selected ligand g-C_3_N_4_/PVP@Ca(OH)_2_, (a) gas phase, (b) aqueous phase.

DFT calculations were employed to assess the electronic properties and global reactivity variables of C_3_N_4_/PVP@Ca(OH)_2_ in both gas and aqueous phase in order to comprehend impact of solvation on its chemical behavior by presenting molecular surface, highlighting orbitals, particularly HOMO and LUMO in Fig. 8. The dipole moment rises from 7.125 D in gas phase to 8.9315 D in aqueous media, indicating enhanced polarity as well as potent interactions with solvent. The regions exhibiting electronegative potential in deep red shade on MESP maps, underscoring areas susceptible to electrophilic and nucleophilic interactions with molecules, which are essential for optimal binding. The oxygen atoms within the g-C_3_N_4_/PVP@Ca(OH)_2_ ring exhibit average Mulliken charges of −0.786286 and −0.809269, suggesting a significant presence of negatively charged regions in associated gas and aqueous phases. The docking results suggest incorporation of oxygen atoms markedly enhances capacity for hydrogen bonding in both hinge and solvent-exposed regions of g-C_3_N_4_/PVP@Ca(OH)_2_. The verdant tone observed on surface indicates an intensified presence of neutral regions, which may have implications for hydrophobic or van der Waals interactions. The observed widening of energy gap in aqueous phase can be ascribed to stabilizing effects of solvent, notably from high polarity of water and its capacity for hydrogen bonding, providing varying degrees of stabilization to both occupied and unoccupied orbitals.

Besides, a slight decrease in aqueous phase, both electronegativity (*χ*) and chemical potential (*μ*) exhibit reduction in electron-attracting capacity and transition towards more stable electronic configuration, also slight increased hardness (0.4385) and reduced softness (0.494 eV^−1^) signify tendency towards electronic rigidity and less chemical reactivity. This aligns with reduction in electrophilicity index, depicting molecule waned potential for electrophilic interactions in aqueous areas (Table 2).

**Table 2:** DFT calculation (quantum chemical descriptors) of the selected ligands

Ligand	Dipole moment (debye)	HOMO (a.u.)	LUMO (a.u.)	Energy gap (Δ*E*_gap_)	Ionization potential (eV)	Electron affinity (eV)	Electronegativity *χ* (eV)	Electrochemical potential *μ* (eV)	Hardness *η* (eV)	Softness *S* (eV^−1^)	Electrophilicity *ω* (eV)
g-C_3_N_4_/PVP@Ca(OH)_2_ (gas)	7.1250	−0.18389	−0.1518	0.872 eV	5.008	4.131	4.570	−4.570	0.4385	1.140	23.82
g-C_3_N_4_/PVP@Ca(OH)_2_ (aqueous)	8.9315	−0.17523	−0.1388	0.98 eV	4.769	3.782	4.276	−4.276	0.494	1.012	18.50

The identified patterns coincide with established impact of solvation on electronic structure. The absence of intermolecular interaction in gaseous state permits orbitals to maintain condition of relative instability. In contrast, the interplay of electrostatic, hydrogen bonding, and dielectric evaluation within aqueous phase enhances orbital stability, particularly for HOMO, thereby reducing availability of electrons for reaction. This behavior retains significance when predicting molecular interactions in biological or polar environments, as it contributes to increased stability in aqueous phase and reduces reactivity. This screening delves deeper into the structural and functional activities, thereby facilitating advancement of nanostructures aimed at enhancing therapeutic efficacy.

## 4. Conclusion

A ternary g-C_3_N_4_/PVP@Ca(OH)_2_ heterostructure was effectively synthesized for the reduction of colored and colorless dyes and antibacterial applications. The crystallite size of Ca(OH)_2_ reduced with the addition of PVP and g-C_3_N_4_, while the surface area increased, which enhanced the exposure of active sites for enhanced antibacterial response. The TEM analysis further confirmed the particle size reduction upon doping. Due to the presence of different types of active sites and large surface area, the 6% g-C_3_N_4_/PVP@Ca(OH)_2_ exhibited 96.4, 91.8, and 36.9% catalytic reduction of RhB, MO and BA dyes in acidic, neutral and basic media, respectively. Furthermore, 6% g-C_3_N_4_/PVP@Ca(OH)_2_ revealed a considerable inhibition domain (9.65 mm) towards *E. coli*. In *silico* predictions agreed with antibacterial activities against *E. coli* and suggested the given nanomaterials as possible inhibitors of DNA Gyrase. The DFT studies, in conjunction with MESP, HOMO, and LUMO analysis, yielded profound insights into the electronic properties and reactivity of active compounds.

In summary, the modified ternary system exhibits excellent performance and can act as ideal catalyst for the reduction of colorful dyes and microbicidal agents.

## Conflicts of interest

No conflict of interest.

## Supplementary Material

RA-015-D5RA03570H-s001

## Data Availability

The data will be made available upon reasonable request. Supplementary information is available. See DOI: https://doi.org/10.1039/d5ra03570h.
