# Supplementary material for: Designing g-C3N4/PVP@Ca(OH)2 ternary heterostructure catalysts for efficient degradation of dyes, antibacterial activity, and molecular docking analysis
Source: RSC Adv. 2025 Sep 15;15(40):33637–47. doi: 10.1039/d5ra03570h (PMC12435568; doi:10.1039/d5ra03570h)
Supplement: RA-015-D5RA03570H-s001 [file RA-015-D5RA03570H-s001.pdf]

Supplementary Information (SI) for RSC Advances.
This journal is © The Royal Society of Chemistry 2025

Designing g-C_3N_4/PVP@$Ca(OH)_2$ ternary heterostructure catalysts for efficient degradation of dyes, antibacterial activity, and molecular docking analysis

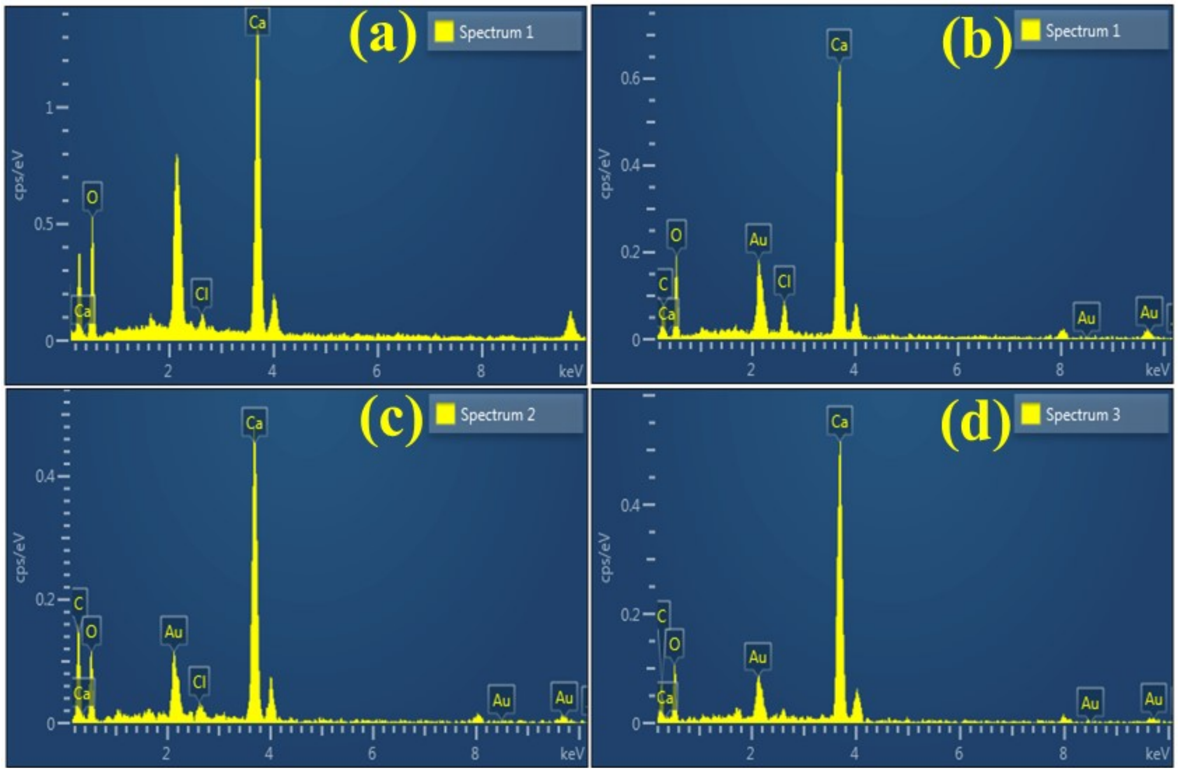

Fig. S1 (a-d) EDS of (a) $Ca(OH)_2$, (b) PVP-$Ca(OH)_2$, (c) 3% g-C_3N_4/PVP-$Ca(OH)_2$, and (d) 6 % g-C_3N_4/PVP-$Ca(OH)_2$

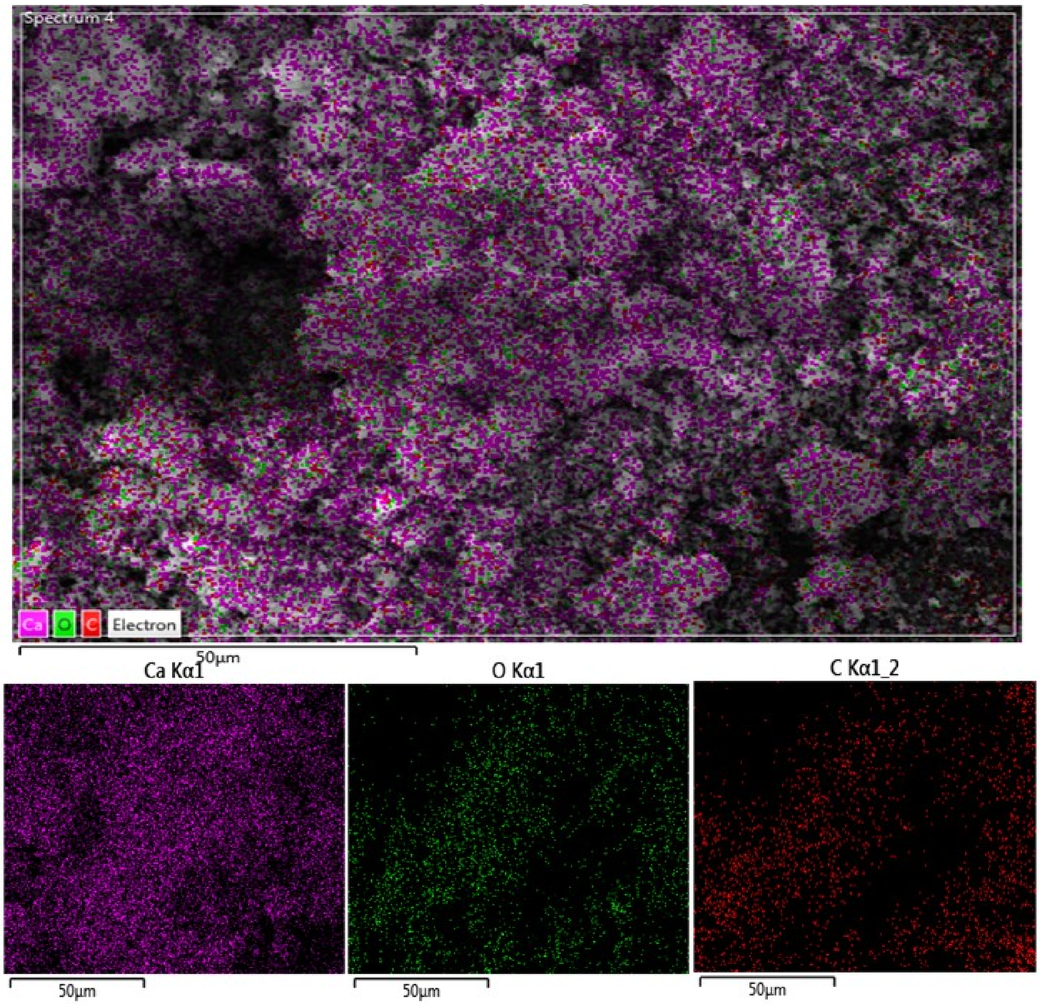

Fig. S2 (a-d) EDS mapping of 6 % g-C_3N_4/PVP-$Ca(OH)_2$

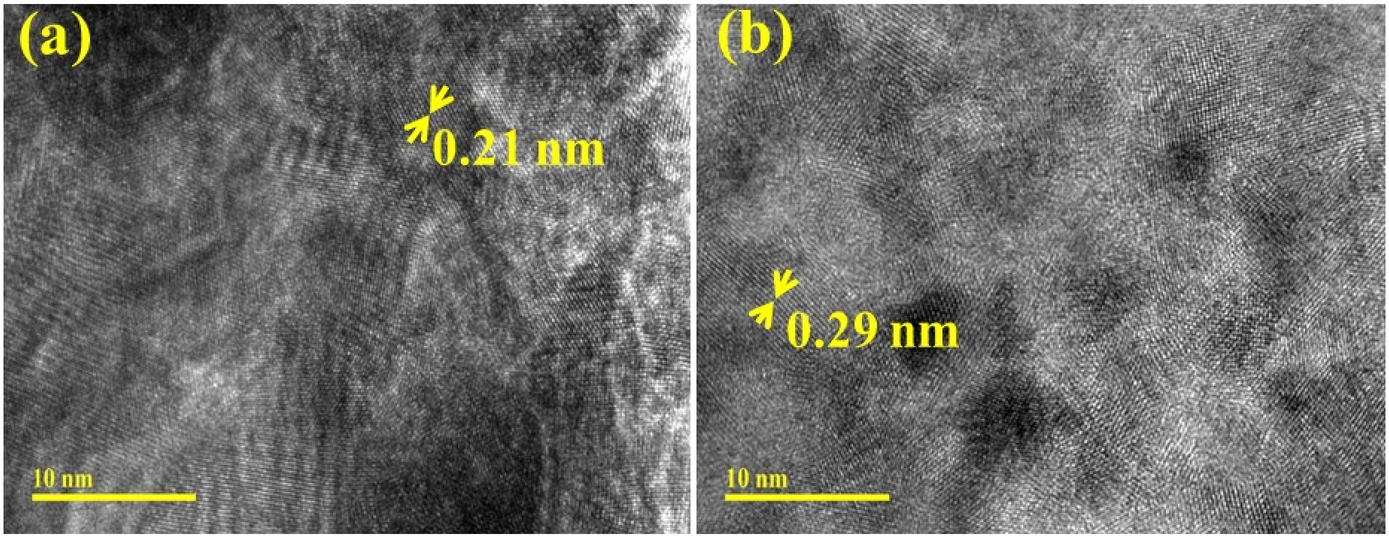

Fig. S3 (a-b) HRTEM images of (a) $Ca(OH)_2$ and (b) 6 % g-C_3N_4/PVP-$Ca(OH)_2$

Table S1: BET surface area of $Ca(OH)_2$ and 6 % g-C_3N_4/PVP- $Ca(OH)_2$

Samples	Correlation Coefficient	BET surface area (m^2/g)
$Ca(OH)_2$	0.9837618	2.1130 ± 0.2757
6 % g-C_3N_4/PVP-$Ca(OH)_2$	0.9941017	2.7305 ± 0.2074

g-C3N_4 doped $Ca(OH)_2$ showed 90.1% degrdation of RhB after 10 minutes (Figure S4)

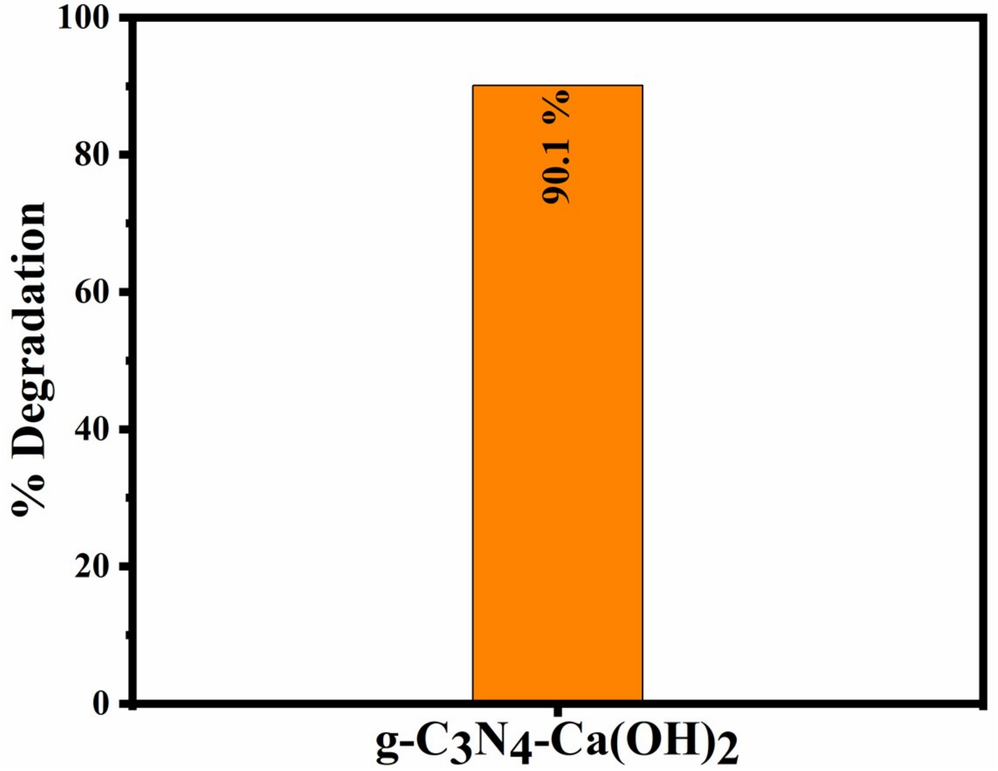

Figure S4: Catalytic activity of g-C_3N_4-$Ca(OH)_2$

Table S2: Comparison of catalytic activity of present work with literature

Catalysts	Synthesis process	Dye	% degradation	References
6% g-C_3N_4/carbon spheres-Bi_2O_3	Co-precipitation	RhB	95.69 (dark)	[1]
Carbon dots/chitosan-La_2O_3	Co-precipitation	RhB	94.57 (in dark)	[2]
N/Fe-TiO_2	Precipitation-hydrothermal	RhB	75 (in light)	[3]
Ag-ZnO	Refluxed chemical method	rose bengal	96 (in light)	[4]
Ag/g-C_3N_4	Biogenic approach	RhB	89 (in light)	[5]
6% g-C_3N_4/PVP-$Ca(OH)_2$	Co-precipitation	RhB	96.4 (in dark)	Present work

Table S3: Comparison of antibacterial activity of present work with literature

Catalysts	Synthesis process	Bacteria	Inhibition zone (mm)	References
6% g-C_3N_4/carbon spheres-Bi_2O_3	Co-precipitation	*E. coli*	2.85	[1]
Carbon dots/chitosan-La_2O_3	Co-precipitation	*E. coli*	4.15	[2]
g-C_3N_4/ eudragit-CdTe	Co-precipitation	*S. aureus*	9.35	[6]
g-C_3N_4	-	*S. aureus*	7	[7]
6% g-C_3N_4/PVP-$Ca(OH)_2$	Co-precipitation	*E. coli*	9.65	Present work

References

[1] Ikram, Muhammad, Muhammad Shazaib, Ali Haider, Anum Shahzadi, Shair Baz, Mohammed M. Algaradah, Anwar Ul-Hamid, Walid Nabgan, Hisham SM Abd-Rabboh, and Salamat Ali. "Catalytic evaluation and in vitro bacterial inactivation of graphitic carbon nitride/carbon sphere doped bismuth oxide quantum dots with evidential in silico analysis." *RSC advances* 13, no. 36 (2023): 25305-25315.

[2] Rasool, Sumaira, Muhammad Imran, Ali Haider, Anum Shahzadi, Walid Nabgan, Iram Shahzadi, Francisco Medina et al. "Efficient dye degradation and antibacterial activity of carbon

dots/chitosan-doped La2O3 nanorods: in silico molecular docking analysis." *Acs Omega* 8, no. 28 (2023): 25401-25409.

[3]. Cong, Ye, Jinlong Zhang, Feng Chen, Masakazu Anpo, and Dannong He. "Preparation, photocatalytic activity, and mechanism of nano-TiO2 co-doped with nitrogen and iron (III)." *The Journal of Physical Chemistry C* 111, no. 28 (2007): 10618-10623.

[4]. Wagh, Snehal S., Vishal S. Kadam, Chaitali V. Jagtap, Dipak B. Salunkhe, Rajendra S. Patil, Habib M. Pathan, and Shashikant P. Patole. "Comparative studies on synthesis, characterization and photocatalytic activity of Ag doped ZnO nanoparticles." *ACS omega* 8, no. 8 (2023): 7779-7790.

[5]. Khan, Mohammad Ehtisham, Thi Hiep Han, Mohammad Mansoob Khan, Md Rezaul Karim, and Moo Hwan Cho. "Environmentally sustainable fabrication of Ag@ g-C3N4 nanostructures and their multifunctional efficacy as antibacterial agents and photocatalysts." *ACS Applied Nano Materials* 1, no. 6 (2018): 2912-2922.

[6]. Aslam, Asma, Muhammad Ikram, Ali Haider, Iram Shahzadi, Anwar Ul-Hamid, Ghafar Ali, Hameed Ullah et al. "Antibacterial potential and dye decolorization with graphitic carbon nitride and eudragit doped CdTe nanostructures evidential molecular docking analysis." *Journal of Photochemistry and Photobiology A: Chemistry* 454 (2024): 115738.

[7]. Sher, Mudassar, Mohsin Javed, Sammia Shahid, Shahid Iqbal, Muhammad Azam Qamar, Ali Bahadur, and Muhammad Abdul Qayyum. "The controlled synthesis of gC 3 N 4/Cd-doped ZnO nanocomposites as potential photocatalysts for the disinfection and degradation of organic pollutants under visible light irradiation." *RSC advances* 11, no. 4 (2021): 2025-2039.
